# Influence of early life stress on depression: from the perspective of neuroendocrine to the participation of gut microbiota

**DOI:** 10.18632/aging.203746

**Published:** 2021-12-10

**Authors:** Xi Tan, Longqing Zhang, Danning Wang, Shaodi Guan, Pei Lu, Xiaolin Xu, Hui Xu

**Affiliations:** 1 Department of Anesthesiology, Tongji Hospital, Tongji Medical College, Huazhong University of Science and Technology, Wuhan, Hubei, China

**Keywords:** early life stress, depression, neuroendocrinology, immune, gut microbiota

## Abstract

Depression is the most common mental disorder and has become a heavy burden in modern society. Clinical studies have identified early life stress as one of the high-risk factors for increased susceptibility to depression. Alteration of the hypothalamic-pituitary-adrenal (HPA) axis in response to stress is one of the key risk factors for depression susceptibility related to early life stress. Laboratory animal studies have demonstrated that maternal separation (MS) for extended periods elicits HPA axis changes. These changes persist into adulthood and resemble those present in depressed adult individuals, including hyperactivity of the HPA axis. In addition, there is growing evidence that inflammation plays an important role in depression susceptibility concerned with early life stress. Individuals that have experienced MS have higher levels of pro-inflammatory cytokines and are susceptible to depression. Recently, it has been found that the gut microbiota plays an important role in regulating behavior and is also associated with depression. The translocation of gut microbiota and the change of gut microbiota composition caused by early stress may be a reason. In this review, we discussed the mechanisms by which early life stress contributes to the development of depression in terms of these factors. These studies have facilitated a systematic understanding of the pathogenesis of depression related to early life stress and will provide new ideas for the prevention and treatment of depression.

## INTRODUCTION

**Figure 1:**
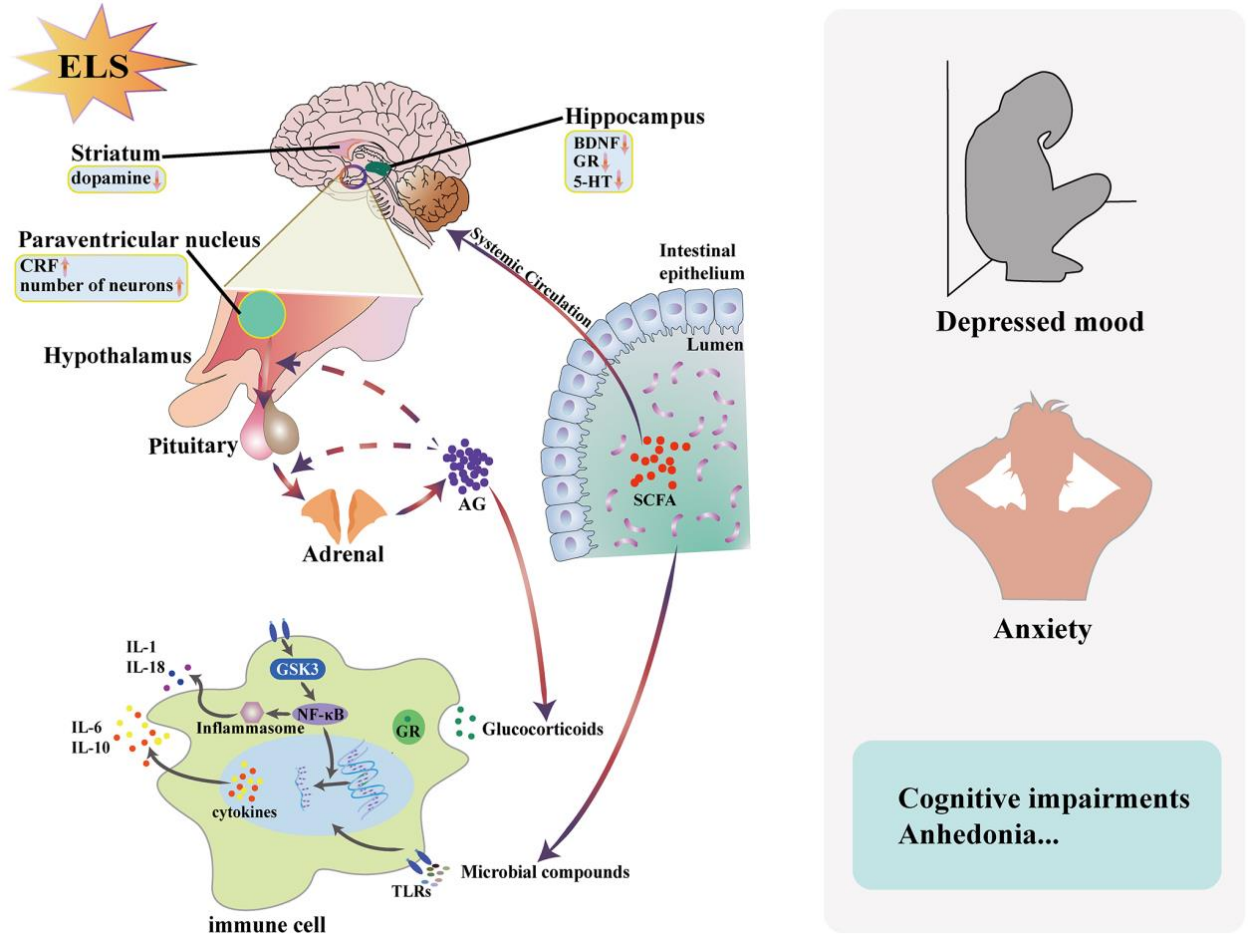
**Early life stress (ELS) contributes to the development of depression through the endocrine system, immune system and gut microbiota.** ELS can lead to high reactivity of HPA axis response to stress and the disorder of HPA axis is closely related to the development of depression. The imbalance of immune system caused by the disorder of glucocorticoid secretion and the change of gut microbiota are also related to the development of depression. Abbreviations: GR: glucocorticoid receptor; TLR: Toll-like receptor; GRF: corticotropin releasing factor; ELS: early life stress; 5-HT: 5-hydroxytryptamine; IL-1: interleukin-1; IL-6: interleukin-6; IL-10: interleukin-10; IL-18: interleukin-18; BDNF, brain-derived neurotrophic factor; SCFA: short-chain fatty acid; AG: adrenal glucocorticoid; GSK3: Glycogen synthase kinase 3; NF-κB: nuclear factor-κ -gene binding.

Clinical studies have found that early life stress is strongly associated with the development of depression in adulthood [1–4]. Individuals that have experienced early life stress have a significantly higher risk of depression, mania, schizophrenia and other psychiatric disorders in adulthood than those have not experienced early life stress [5]. Early life stress is significantly more associated with the onset of depression than recent stressful events with depression [6]. In addition, patients with depression who experienced a stressful event in childhood showed clinical features such as earlier age of onset, more severe depressive symptoms, more prolonged course, recurrent episodes and significantly reduced efficacy of common antidepressants [7]. Basic research has also shown that early environmental stress induces depression in rodents and primates in adulthood [8]. However, the pathways or mechanisms through which stressful events occurring early in life contribute to the onset of depression are not yet entirely clear. Changes in the HPA axis are thought to be key factors in depression susceptibility [9]. For example, MS can produce significant stress. Long-term exposure to stress will lead to the HPA axis hyperactivity in response to stress, which manifests itself in adulthood and persists. In addition, MS increases susceptibility to depression by altering both serotonin and dopamine secretion in relevant areas of the brain. Besides the endocrine system, MS is also closely linked to the immune system. Inflammatory mechanisms are involved in enhancing the stress response after early life stress and in the development of vulnerability to depression [10, 11]. Not only that, recent studies have found that the altered gut microbiota caused by MS may also play an important role. Alterations in composition have been found in MS models and rat models of depression. Even fecal transplants from Major Depressive Disorder (MDD) patients affected depression-like behavior in recipient animals [12]. As shown in Figure 1, we demonstrated the association between early-life stress and depression from the perspective of neuroendocrinology, immune and gut microbiota.

## Neuroendocrine regulation in early life stress-induced depression

In rodents (especially rats), MS has become a common trigger for various psychiatric disorders, especially depression [13–17]. MS may contribute to depression susceptibility in adulthood through alterations of the HPA axis in response to stress [18, 19], alterations in the expression of BDNF in different regions of the central nervous system (CNS) [20], alterations in the expression of serotonin in the CNS [21] and alterations in the expression of dopamine and its receptors [22] (Table 1).

**Table 1:** Neuroendocrine regulation in early life stress-induced depression.

**System involved**	**Stress exposure**	**Depression-like behavior**	**Main findings**	**References**
HPA axis	MS	Promoting	Venlafaxine reverses depressive-like behavior induced by MS via modulating HPA axis activity.	Martisova et al., 2015
	MS	Promoting	Acupuncture reverses depressive-like behavior induced by MS via modulating HPA activity.	Park et al., 2011
Serotonin system	MS	Promoting	Serotonergic activity in the hippocampus and the raphe decrease under MS-induced depression.	Jahng, 2011
	MS	Promoting	5-HT synthesis in hippocampal dentate gyrus decreases in the MS rat pups.	Baek et al., 2012
	ELS	Promoting	The ELS-induced decrease of SERT expression relates to altered serotonergic function, and possibly to the susceptibility to depression.	Wankerl et al., 2014
Dopamine system	MS	Promoting	Down regulation of D1 receptors promotes the depression-like behavior caused by MS.	Amiri et al., 2016
Neurotrophins	MS	Promoting	Enriched environment during the early development period is effective in alleviating depression induced by ELS through increasing BDNF expression in the hippocampus.	Huang et al., 2021
	MS	Promoting	Through the BDNF/PKA/CREB pathway, SiNiSan treatment might impose antidepressant effects on young and adult MS rats.	Cao et al., 2019

Abbreviations: MS: maternal separation; HPA axis: Hypothalamic–pituitary–adrenal axis; ELS: early life stress; 5-HT: 5-hydroxytryptamine; CREB: cAMP-response element binding protein; BDNF: brain-derived neurotrophic factor; PKA: protein kinase A.

### HPA axis

Studies over the past few decades have demonstrated that hyperactivity of the HPA axis is one of the most consistent biological findings in depression [23–27]. Laboratory animal studies have shown that separating neonatal rodents and non-human primates from their mothers for long periods elicits HPA axis changes. Those changes persist into adulthood and resemble those present in depressed adult individuals, including hyperactivity of the HPA axis [28].

Male Wistar rats underwent early MS showed significantly reduced glucocorticoid receptor density in the hippocampus in adulthood and exhibited depression-like behaviors in the forced swim test in adulthood [29]. Adult mice also showed lasting consequences of ELS using limited nesting and bedding material paradigm including HPA axis hyperreactivity [30]. Venlafaxine reversed the deleterious effects of chronic stress including stress-induced depression-like behaviors and cognitive deficits. Besides, it reduced subventricular zone volume, demonstrating that modulation of stress-mediated glucocorticoid secretion may be a target for the treatment of mood disorders and neurodegenerative processes [31]. Acupuncture therapies from the East also appeared to improve MS. The HPA axis reactivity was mitigated by acupuncture, specifically by reducing CORT and ACTH plasma levels in MS rats [32]. When endogenous glucocorticoid level is high, GR is more important in regulating HPA axis [33]. In the case of elevated circulating cortisol levels, depressed patients show impaired HPA negative feedback. Many studies have described the decrease of GR function (GR resistance) in patients with depression and concluded that antidepressants play a role by reversing these hypothetical GR changes [34]. When the stress improves over time, depression behavior can be improved to some extent.

### Neurotrophins

Not only does MS alter the response of the HPA axis to stress, but MS has also been found to cause alterations in neurotransmitters and brain-derived neurotrophic factor (BDNF) in the brain [35–39], a neurotrophic factor expressed in the brain and associated with neuronal growth, synaptic plasticity, differentiation and neuronal survival [40]. MS decreased hippocampal BDNF and p-AKT/AKT levels and was associated with depression-like behavior, while an enriched environment reversed this negative impact and upregulated the PI3K-AKT pathway [41]. Direct infusion of BDNF into the hippocampus or midbrain yielded antidepressant-like effects [42]. BDNF was also required for the rapid antidepressant effects of ketamine [43]. Some findings indicated that fast transient translation of BDNF was necessary for ketamine’s fast-acting and long-lasting antidepressant-like behavioral effects. Those long-term antidepressant responses may be due to alterations in synaptic plasticity initiated by transient increases in BDNF translation [44]. Eukaryotic elongation factor 2 kinase (eEF2K) null knockout mice administered an acute low dose of ketamine did not have increased BDNF protein expression and did not show an antidepressant response to the drug [45].

### Serotonin system

In addition to BDNF, serotonin plays an important role in MS-induced depression. Serotonin is closely associated with mood disorders and plays a role in the corticolimbic network that regulates mood, behavior, cognition and motor function. Serotonin transporter gene (SERT) DNA methylation is thought to be related to stress-related diseases. [46, 47]. In mice with MS, 5-hydroxytryptamine (5-HT) synthesis in the dorsal suture nucleus and cell proliferation in the hippocampal dentate gyrus were significantly reduced [48]. Depression-like behavior was also observed in two-month-old MS rats with reduced 5-HT activity in the hippocampus [49]. SERT gene (Solute Carrier Family 6 Member 4, SLC6A4) encodes a protein that transports the neurotransmitter serotonin from the synaptic gap to presynaptic neurons. It has been shown that ELS caused SLC6A4 methylation and that reduced SLC6A4 expression allowed serotonin to accumulate in the synaptic gap [50], thereby impairing normal serotonin function and leading to depression. Besides, MS triggered the decrease in 5-HT1_A_ receptor expression in the CA1 region in the hippocampus of young and adult male rats compared with control rats without MS, which is also a key factor for depression [20].

Besides 5-HT, a dopaminergic pathway is a part of the reward system. Due to the interaction between the dopaminergic system and HPA axis or the interaction between the dopaminergic system and serotonin system, the effect of chronic stress on reward perception may lead to depression. Some studies have demonstrated that early psychological stress activates the HPA axis, exacerbates DA depletion and is associated with a decrease in DA synthesis in the brain. DA deficiency resulting from early life stress may, in some instances, predispose an individual to depression [51]. In addition, MS causes depression-like behavior in adult male mice with reduced dopamine level in the striatum. A drug that blocks the metabolism of dopamine, selegiline, reduces depression-like behavior in MS mice. Both dopamine receptors D1 and D2 mediate the antidepressant-like effects of selegiline, with D1 receptors mediating the effects on depression behavior and D2 receptors mediating the effects on pleasure deficit [52].

## Immunomodulation as a key role in early life stress-induced depression

HPA axis and inflammation modulation under early life stress have a close relationship. Both psychogenic and immune stressors can induce HPA axis and inflammation changes. Maternal care deprivation (a psychological stressor) model increased the levels of pro-inflammatory cytokines (interleukin-1β (IL-1β), interleukin-6 (IL-6) and tumor necrosis factor-α (TNF-α)) and decreased the anti-inflammatory cytokine (interleukin-10) level in the brain and serum throughout developmental programming [53]. Meanwhile, early exposure to lipopolysaccharide (an immune stressor) elevated the levels of TNF-α and IL-1β in the hippocampus in adulthood and also increased corticosterone levels in adulthood [54]. There is a growing awareness that people with autoimmune disorders show a high prevalence of depression disorders. By the early 1990 s, the role of overproduction of immunomodulatory signaling molecules for depression became apparent, particularly pro-inflammatory cytokines, which may play a role in the development and maintenance of depression [55]. Interleukin-1 (IL-1), interferon-gamma (IFN-γ), acute-phase associated proteins and tumor cytokines have now been reported to be associated with depression disorder [56]. In addition, treating the hepatitis C virus with pro-inflammatory agents such as interferon-alpha (IFN-α) leads to depression symptoms in a quarter of patients [57]. This inflammatory phenotype is also thought to be an important factor in treatment resistance in depression. This theory led researchers to investigate the antidepressant effects of anti-inflammatory compounds and showed that TNF antagonism improved depressive symptoms in patients with high baseline inflammatory biomarkers [58]. Given that many antidepressants have anti-inflammatory effects [59], immune mechanisms are now thought to be central to the development of depressive symptoms.

Studies have suggested that inflammation plays a key role in early life stress leading to depression susceptibility. Related research results are listed in Table 2. Studies have also showed that repeated MS has pro-inflammatory immune consequences in diverse tissues. Repeated MS animals exhibited greater microglial activation and elevated pro-inflammatory cytokine signaling in key brain regions implicated in human psychiatric disorders. A recent review indicated that minocycline inhibited microglial activation and alleviated depression-like behaviors in male adolescent mice subjected to MS [60]. A prospective study showed that depression adults who experienced severe early life stress were 1.48 times more likely to have clinically high C-reactive protein (CRP) levels than those depression adults without early life stress [61]. In a study that followed adolescent females at higher risk of depression for more than 2.5 years study, adolescents with a history of early life stress had greater increases in IL-6 and CRP when they became depressed than their peers without a history of early life stress. In addition, in this study, adolescents without a history of early life stress had lower CRP as depressive symptoms decreased. In contrast, adolescents with a history of early life stress did not have this association [62]. There is evidence that the link between early life stress, inflammation and depression is detectable at a young age. A longitudinal study found that patients who experienced early life stress followed by depression had significantly higher CRP level than those who only suffered from depression and did not experience early life stress [63]. Early life stress increased expression of the gene encoding Toll-like receptor (TLR) 4, which activates the innate immune system response. Besides, early life stress reduced the gene expression encoding the glucocorticoid receptor, responsible for down-regulating inflammation in the cortisol response [64]. The above studies have suggested that inflammation plays a vital role in early life stress leading to depression susceptibility.

**Table 2:** Immunomodulation as a key role in early life stress-induced depression.

**Inflammatory cytokine**	**Stress exposure**	**Depression-like behavior**	**Main findings**	**References**
IL-1β	MS	Promoting	IL-1β in the vHIP, PFC and serum increase under ELS-induced depression	Wang et al., 2017
IL-6	MS	Promoting	IL-6 increases under ELS-induced depression.	Miller and Cole, 2012
Il-10	MS	Promoting	IL-10 in the amygdala and hypothalamus decrease under ELS-induced depression.	DellaGioia et al., 2010
TNF-α	MS	Promoting	Pro-inflammatory markers TNF-α is up regulated under ELS-induced depression.	Wang et al., 2017
CRP	ELS	Promoting	CRP increases under ELS-induced depression.	Danese et al., 2008
TLR 4	ELS	Promoting	The expression of gene encoding TLR 4 is up-regulated under ELS-induced depression.	Carroll et al., 2011

Abbreviations: IL-1β: interleukin-1β; MS: maternal separation; vHIP: ventral hippocampus; PFC: prefrontal cortex; IL-6: interleukin-6; IL-10: interleukin-10; TNF-α: tumor necrosis factor-α; CRP: C-reactive protein; ELS: early life stress; TLR 4: Toll-like receptor.

Studies have shown a sustained process of MS increased inflammatory cytokine secretion in peripheral and brain tissue in mice exposed to lipopolysaccharide (LPS) as adults [65]. Expression of the neuroinflammatory marker Iba1 was increased in MS mice [66]. MS also induced depression-like behavior with microglia activation and over expression of histone demethylase Jumonji domain-containing protein 3 (Jmjd3). These changes can also be found in adulthood. Jmjd3, a trimethylated lysine 27 in histone 3 (H3K27me3) demethylase, can be activated by nuclear factor-kappa B (NF-κB), further regulating the expression of pro-inflammatory cytokines and resulting in neuroinflammation. Treatment with the demethylase Jmjd3 inhibitor GSK-J4 attenuated these changes, suggesting that Jmjd3 is involved in MS-induced depression susceptibility by enhancing neuroinflammation in the rat prefrontal cortex and hippocampus [67]. It has been shown that depression-like behavior following MS stress is associated with increased expression of TLR-4 and its main signaling protein Myd88 in the hippocampus. Voluntary physical activity during adolescence can prevent the negative effect of early life stress. The depression effects of stress are mediated, at least in part, by attenuating the innate immune response in the hippocampus [68]. MS upregulated pro-inflammatory markers TNF-α and downregulated anti-inflammatory markers IL-10 in the hippocampus, which activated microglia and promoted pro-inflammatory shifts in microglia [69]. Early life stress reduced IL-10 expression in the amygdala and hypothalamus [56], and these effects could be reversed by minocycline [70]. Besides, MS increased depression and anxiety behavior with an increased level of IL-1β in the ventral hippocampus (vHIP), prefrontal cortex (PFC) and serum, a decreased level of IL-10 in HPV [69]. Fluvoxamine had similar effects with mRNA levels of IL-1β, IL-6 and TNF-α downregulated in the striatum of fluvoxamine-treated rats. Early treatment with fluvoxamine suppressed depression behavior in MS mice by promoting the expression of anti-inflammatory cytokines [71]. It has been shown that injection of live and heat-killed PS23 cells showed positive behavioral effects in MS animals with increased propensity to explore and activity in behavioral tests and reduced anxiety and depression.

## Role of gut microbiota in early life stress-induced depression

### Investigation of gut microbiota

In addition to HPA axis and the immune system, gut microbiota shaped by early life stress increased susceptibility to depression in adulthood. The microbiota-gut-brain axis refers to the two-way communication between the gut microbiota and the brain. Although not fully understood, this complex interaction involves multiple physiological systems such as the gastrointestinal system and its gut microbiota, nervous systems, the immune system and the neuroendocrine system [72]. The gut microbiota has emerged as an important brain and behavior regulator linked to depression [73]. Maes et al. suggested that gut microbiota translocation or leaky gut may be a major trigger for the development of depression. Gut microbiota translocation or leaky gut can activate immune cells and stimulate selective immunoglobulin A (IgA) and immunoglobulin M (IgM), which indicating that gut microbiota may be involved in the pathophysiology of depression by causing a progressive immune response [74]. In addition, recent studies have shown that the gut microbiota regulate the maturation of microglia, possibly through the serotonin pathway or the secretion of metabolites such as short-chain fatty acids (SCFA) [75].

Meanwhile, early life stresses, such as maternal immune activation and MS, have been shown to produce gut defects such as increased gut permeability, which lead to translocation of gut microbiota [76]. Therefore, it can be speculated that altered gut permeability and translocation of gut microbiota due to early life stress are closely linked to subsequent depression episodes. Besides, early life stress shaped the gut microbiota and was associated with disease in later adulthood [77, 78]. Chronic exposure to limited nesting stress during the first-week postnatally has sustained effects monitored at weaning including hypercorticosteronemia, a leaky gut and a decreased gut microbiota diversity [79]. However, the underlying mechanisms by which stress regulates microbial community composition remain to be elucidated. For example, a large body of evidence suggested that depression is associated with alterations in the gut microbiota composition, often manifested as a reduction in abundance and diversity, fecal microbiota transplantation is expected to treat diseases related to intestinal gut microbiota disorder. [12, 80]. 16S rRNA analysis of fecal samples from healthy individuals revealed that the most abundant bacteria in terms of numbers were phylum *Aspergillus*, accounting for 70–75% of the entire, with other bacteria also included phylum *Aspergillus*, phylum *Actinomycetes*, phylum *Clostridium* and phylum *Verrucomicrobial* [81]. The proportional number of microbiota differed in depression patients. Compared to healthy people, the largest numbers were found in the phylum *Anaplasma* and lower numbers in the family *Lachnospiraceae* [82]. Similar studies in 2015 found that patients with depression had higher levels of *Bacteroides*, *Proteus* and *Acinetobacter*, while the number of *Firmicutes* was significantly lower [83].

Recently, researchers used DNA sequencing to analyze the microbiota in the feces of more than 1000 people in the Flemish gut microbiota in Belgium found that *Coprococcus* and *Dialisterwere* reduced in patients with depression. There was a positive correlation between their quality of life and the potential ability of the gut microbiota to synthesize 3,4-dihydroxyphenylacetic acid, a breakdown product of the neurotransmitter dopamine [84]. These results were the strongest evidence to date that a person’s microbiota can influence their mental health. Analysis of the gut microbiota of MS rats revealed alterations in the composition of their gut microbiota. Member of the *actinomycetes* was reduced, while the abundance of member of the *proteobacteria* was elevated [83].

### Therapeutic potential of probiotics

**Figure 2:**
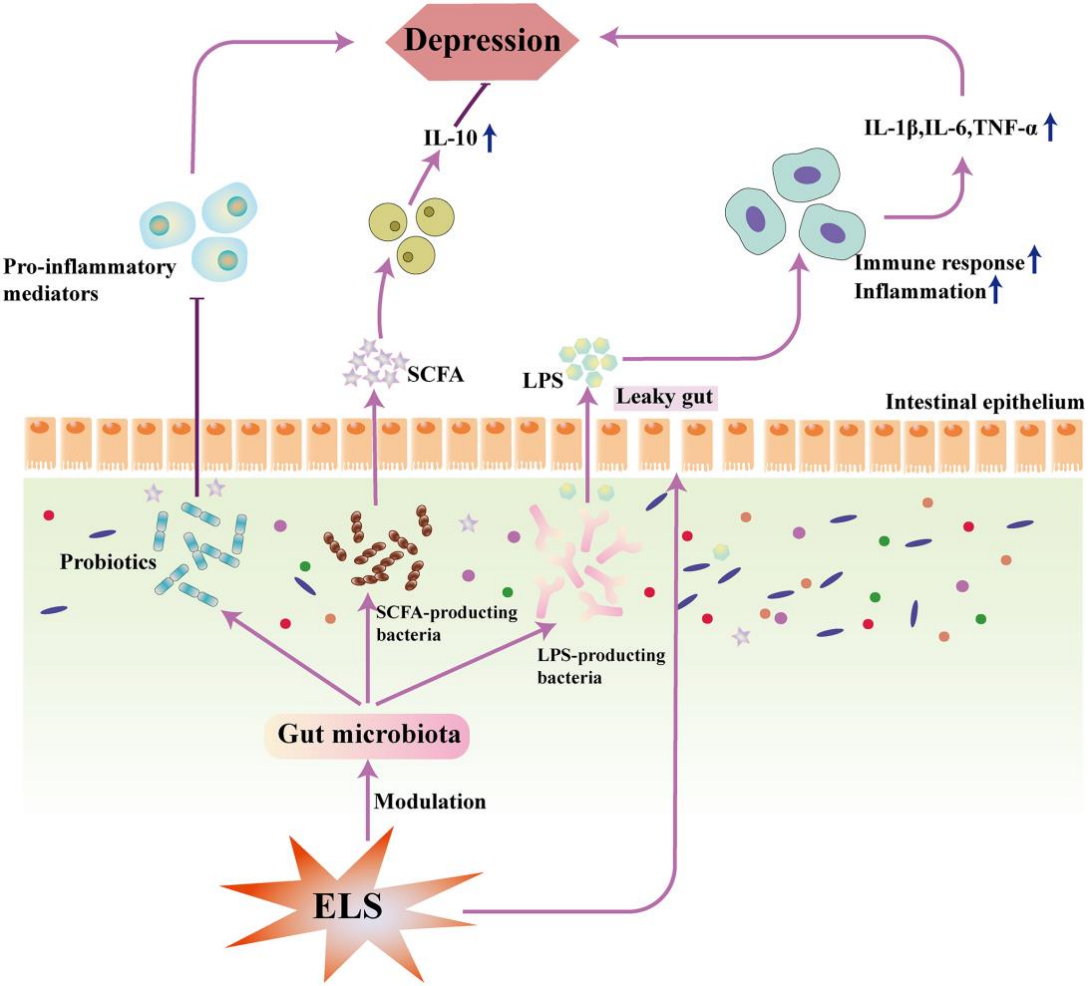
**Early life stress produces gut defects and increases gut permeability, leading to translocation of LPS and gut microbiota.** LPS can aggravate the body’s inflammatory response and increase the risk of depression. Probiotics and SCFA can reverse this process and reduce the risk of depression. Abbreviations: LPS: lipopolysaccharide; SCFA: short-chain fatty acid; ELS: early life stress; IL-1β: interleukin-1β; IL-6: interleukin-6; IL-10: interleukin-10; TNF-α: tumor necrosis factor-α.

Probiotic interventions have also been shown to reduce depression-like behavior in rats and mice and improve inflammatory responses (Table 3). In addition, some probiotics have now been found to reverse early life stress-induced gut microbiota disturbances and persistent activation of the HPA axis. Eicosapentaenoic acid/Docosahexaenoic (EPA/DHA) acid treatment normalizes the interference of early life stress on gut microbiota in female rats. The altered composition of the gut microbiota resulted in reduced levels of gut permeability and thus reduced inflammation [85]. *Bifidobacterium* *pseudocatenulatum* *CECT7765* can also ameliorate MS-induced gut inflammation (decreased interferon-gamma, IFN-γ), which improves depression-like behaviors [86]. As shown in Figure 2, early stress can affect depression through gut microbiota. *Bifidobacterium (B.) bifidum G9-1* prevents MS-induced hypercortisolemia, reduces MS-induced high corticosterone level [87]. Bifidobacterium infantis decreased depression-like behavior in MS mice in forced swimming and sucrose preference test [88]. Mice administered with live *Lactobacillus paracasei PS23 (PS23)* cells had lower serum corticosterone levels and higher serum anti-inflammatory interleukin-10 (IL-10) levels, suggesting that the effect of probiotics may be associated with immunomodulatory properties. Ingestion of *PS128* ameliorated depression-like behaviors and modulated neurochemicals related to affective disorders [89]. The study demonstrated the potential of *PS23* cells in reversing abnormalities induced by early life stress [90]. Interestingly, both live and heat-killed *PS23* also reversed anxiety-like and depression-like behaviors induced by chronic corticosterone administration in mice [91].

**Table 3:** Role of gut microbiota in early life stress-induced depression.

**Probiotics**	**Stress exposure**	**Depression-like behavior**	**Main findings**	**Reference**
Bifidobacterium pseudocatenulatum CECT7765	MS	Promoting	B. pseudocatenulatum CECT 7765 administration reduces depression-like behavior in adulthood, reverses intestinal dysbiosis and reduces corticosterone production.	Moya-Pérez et al., 2017
Bifidobacteria	MS	Promoting	Bifidobacteria treatment results in normalization of immune response and reversal of behavioral deficits.	Fukui et al., 2018
Lactiplantibacillus plantarum PS128	MS	Promoting	Ingestion of PS128 ameliorates depression-like behaviors and modulates neurochemicals.	Liu YW al et al., 2016
Lactobacillus paracasei PS23	MS	Promoting	PS23 cells decrease serum corticosterone levels accompanied by higher serum anti-inflammatory IL-10 levels with reducing depression-like behavior.	Liao et al., 2019
Heat-killed Lactobacillus paracasei PS23	ELS	Promoting	PS23 reverses ELS-induced depression-like behaviors.	Wei et al., 2019

Abbreviations: MS: maternal separation; ELS: early life stress.

The current research is mainly based on specific gut microbiota, their metabolites and neurological symptoms. But these correlations do not prove cause and effect. Besides, many studies have used animal models that do not accurately reflect human characteristics or behavior. Studies on humans are relatively few. They are usually based on relatively small populations that may not control for many confounding factors that may affect the gut microbiota, such as abnormal diets, antibiotics or antidepressants. The relationship between early stress and the interaction of gut microbiota and its metabolites deserves to be further studied to find a new therapeutic target to reduce the negative effects of early stress.

## CONCLUSIONS

The delicate balance between the stress response, immunity and gut microbiota is crucial for nervous system health. Early life stress can result in the dysregulation of brain physiology and behavior, contributing to the development of depression. More cohort studies are needed to further reveal the effect of early life stress on adulthood. Till now, how to reduce the effect of early life stress on adulthood is also a problem worthy of study. Meanwhile, much work is also needed at a mechanistic level in preclinical and human studies to tease apart the relative contribution of each of them and the cross-talk between each other.

As stated in the introduction, we now appreciate that imbalanced stress and inflammatory responses induced by early life stress are undoubtedly involved in the development and maintenance of depression, but recent evidence suggests that the gut microbiota, too, may play a role in the imbalance of these pathways and neuropsychology. The role of the microbiota in disease is only now emerging, particularly in the field of neuropsychology. Further studies may focus on the cross-talk between the microbiota and neuroendocrine or immune system under early life stress and related intervention therapy.

## References

[1] Kessler RC, McLaughlin KA, Green JG, Gruber MJ, Sampson NA, Zaslavsky AM, Aguilar-Gaxiola S, Alhamzawi AO, Alonso J, Angermeyer M, Benjet C, Bromet E, Chatterji S, et al. Childhood adversities and adult psychopathology in the WHO World Mental Health Surveys. Br J Psychiatry. 2010; 197:378–85. 10.1192/bjp.bp.110.080499 21037215 PMC2966503

[2] Mandelli L, Petrelli C, Serretti A. The role of specific early trauma in adult depression: A meta-analysis of published literature. Childhood trauma and adult depression. Eur Psychiatry. 2015; 30:665–80. 10.1016/j.eurpsy.2015.04.007 26078093

[3] LeMoult J, Humphreys KL, Tracy A, Hoffmeister JA, Ip E, Gotlib IH. Meta-analysis: Exposure to Early Life Stress and Risk for Depression in Childhood and Adolescence. J Am Acad Child Adolesc Psychiatry. 2020; 59:842–55. 10.1016/j.jaac.2019.10.011 31676392 PMC11826385

[4] Targum SD, Nemeroff CB. The Effect of Early Life Stress on Adult Psychiatric Disorders. Innov Clin Neurosci. 2019; 16:35–37. 31037228 PMC6450674

[5] Agid O, Shapira B, Zislin J, Ritsner M, Hanin B, Murad H, Troudart T, Bloch M, Heresco-Levy U, Lerer B. Environment and vulnerability to major psychiatric illness: a case control study of early parental loss in major depression, bipolar disorder and schizophrenia. Mol Psychiatry. 1999; 4:163–72. 10.1038/sj.mp.4000473 10208448

[6] Gilmer WS, McKinney WT. Early experience and depressive disorders: human and non-human primate studies. J Affect Disord. 2003; 75:97–113. 10.1016/s0165-0327(03)00046-6 12798250

[7] Tennant C, Smith A, Bebbington P, Hurry J. Parental loss in childhood: relationship to adult psychiatric impairment and contact with psychiatric services. Arch Gen Psychiatry. 1981; 38:309–14. 10.1001/archpsyc.1981.01780280077009 7212962

[8] Pryce CR, Rüedi-Bettschen D, Dettling AC, Weston A, Russig H, Ferger B, Feldon J. Long-term effects of early-life environmental manipulations in rodents and primates: Potential animal models in depression research. Neurosci Biobehav Rev. 2005; 29:649–74. 10.1016/j.neubiorev.2005.03.011 15925698

[9] Björkenstam E, Vinnerljung B, Hjern A. Impact of childhood adversities on depression in early adulthood: A longitudinal cohort study of 478,141 individuals in Sweden. J Affect Disord. 2017; 223:95–100. 10.1016/j.jad.2017.07.030 28735168

[10] Kuhlman KR, Robles TF, Haydon MD, Dooley L, Boyle CC, Bower JE. Early life stress sensitizes individuals to the psychological correlates of mild fluctuations in inflammation. Dev Psychobiol. 2020; 62:400–408. 10.1002/dev.21908 31489628 PMC8211401

[11] Brenhouse HC, Danese A, Grassi-Oliveira R. Neuroimmune Impacts of Early-Life Stress on Development and Psychopathology. Curr Top Behav Neurosci. 2019; 43:423–47. 10.1007/7854_2018_53 30003509

[12] Kelly JR, Borre Y, O' Brien C, Patterson E, El Aidy S, Deane J, Kennedy PJ, Beers S, Scott K, Moloney G, Hoban AE, Scott L, Fitzgerald P, et al. Transferring the blues: Depression-associated gut microbiota induces neurobehavioural changes in the rat. J Psychiatr Res. 2016; 82:109–18. 10.1016/j.jpsychires.2016.07.019 27491067

[13] Zheng Y, He J, Guo L, Yao L, Zheng X, Yang Z, Xia Y, Wu X, Su Y, Xu N, Chen Y. Transcriptome Analysis on Maternal Separation Rats With Depression-Related Manifestations Ameliorated by Electroacupuncture. Front Neurosci. 2019; 13:314. 10.3389/fnins.2019.00314 31024237 PMC6460510

[14] Park SW, Seo MK, Lee JG, Hien LT, Kim YH. Effects of maternal separation and antidepressant drug on epigenetic regulation of the brain-derived neurotrophic factor exon I promoter in the adult rat hippocampus. Psychiatry Clin Neurosci. 2018; 72:255–65. 10.1111/pcn.12609 28990703

[15] Lee JM, Kim TW, Park SS, Kim CJ, Shin MS, Lee SJ, Kim SH, Baek SS. Wnt signaling pathway is implicated in the alleviating effect of treadmill exercise on maternal separation-induced depression. J Exerc Rehabil. 2019; 15:200–05. 10.12965/jer.1938148.074 31111001 PMC6509450

[16] Marais L, van Rensburg SJ, van Zyl JM, Stein DJ, Daniels WM. Maternal separation of rat pups increases the risk of developing depressive-like behavior after subsequent chronic stress by altering corticosterone and neurotrophin levels in the hippocampus. Neurosci Res. 2008; 61:106–12. 10.1016/j.neures.2008.01.011 18329744

[17] Vetulani J. Early maternal separation: a rodent model of depression and a prevailing human condition. Pharmacol Rep. 2013; 65:1451–61. 10.1016/s1734-1140(13)71505-6 24552992

[18] Aisa B, Tordera R, Lasheras B, Del Río J, Ramírez MJ. Effects of maternal separation on hypothalamic-pituitary-adrenal responses, cognition and vulnerability to stress in adult female rats. Neuroscience. 2008; 154:1218–26. 10.1016/j.neuroscience.2008.05.011 18554808

[19] Amini-Khoei H, Haghani-Samani E, Beigi M, Soltani A, Mobini GR, Balali-Dehkordi S, Haj-Mirzaian A, Rafieian-Kopaei M, Alizadeh A, Hojjati MR, Validi M. On the role of corticosterone in behavioral disorders, microbiota composition alteration and neuroimmune response in adult male mice subjected to maternal separation stress. Int Immunopharmacol. 2019; 66:242–50. 10.1016/j.intimp.2018.11.037 30500621

[20] Cao K, Shen C, Yuan Y, Bai S, Yang L, Guo L, Zhang R, Shi Y. SiNiSan Ameliorates the Depression-Like Behavior of Rats That Experienced Maternal Separation Through 5-HT1A Receptor/CREB/BDNF Pathway. Front Psychiatry. 2019; 10:160. 10.3389/fpsyt.2019.00160 30984042 PMC6447714

[21] Park SS, Park HS, Kim CJ, Baek SS, Kim TW. Exercise attenuates maternal separation-induced mood disorder-like behaviors by enhancing mitochondrial functions and neuroplasticity in the dorsal raphe. Behav Brain Res. 2019; 372:112049. 10.1016/j.bbr.2019.112049 31229645

[22] Fone KC, Porkess MV. Behavioural and neurochemical effects of post-weaning social isolation in rodents-relevance to developmental neuropsychiatric disorders. Neurosci Biobehav Rev. 2008; 32:1087–102. 10.1016/j.neubiorev.2008.03.003 18423591

[23] Pariante CM, Lightman SL. The HPA axis in major depression: classical theories and new developments. Trends Neurosci. 2008; 31:464–68. 10.1016/j.tins.2008.06.006 18675469

[24] Jokinen J, Nordström P. HPA axis hyperactivity as suicide predictor in elderly mood disorder inpatients. Psychoneuroendocrinology. 2008; 33:1387–93. 10.1016/j.psyneuen.2008.07.012 18805641

[25] Zhu LJ, Liu MY, Li H, Liu X, Chen C, Han Z, Wu HY, Jing X, Zhou HH, Suh H, Zhu DY, Zhou QG. The different roles of glucocorticoids in the hippocampus and hypothalamus in chronic stress-induced HPA axis hyperactivity. PLoS One. 2014; 9:e97689. 10.1371/journal.pone.0097689 24831808 PMC4022669

[26] Jokinen J, Nordström P. HPA axis hyperactivity and attempted suicide in young adult mood disorder inpatients. J Affect Disord. 2009; 116:117–20. 10.1016/j.jad.2008.10.015 19054569

[27] Wang S, Wang C, Yu Z, Wu C, Peng D, Liu X, Liu Y, Yang Y, Guo P, Wei J. Agarwood Essential Oil Ameliorates Restrain Stress-Induced Anxiety and Depression by Inhibiting HPA Axis Hyperactivity. Int J Mol Sci. 2018; 19:3468. 10.3390/ijms19113468 30400578 PMC6274913

[28] Sánchez MM, Ladd CO, Plotsky PM. Early adverse experience as a developmental risk factor for later psychopathology: evidence from rodent and primate models. Dev Psychopathol. 2001; 13:419–49. 10.1017/s0954579401003029 11523842

[29] Ferrara N, Clapp C, Weiner R. The 16K fragment of prolactin specifically inhibits basal or fibroblast growth factor stimulated growth of capillary endothelial cells. Endocrinology. 1991; 129:896–900. 10.1210/endo-129-2-896 1855480

[30] McIlwrick S, Rechenberg A, Matthes M, Burgstaller J, Schwarzbauer T, Chen A, Touma C. Genetic predisposition for high stress reactivity amplifies effects of early-life adversity. Psychoneuroendocrinology. 2016; 70:85–97. 10.1016/j.psyneuen.2016.04.023 27179233

[31] Martisova E, Aisa B, Tordera RM, Puerta E, Solas M, Ramirez MJ. Venlafaxine reverses decreased proliferation in the subventricular zone in a rat model of early life stress. Behav Brain Res. 2015; 292:79–82. 10.1016/j.bbr.2015.05.059 26051818

[32] Park HJ, Park HJ, Chae Y, Kim JW, Lee H, Chung JH. Effect of acupuncture on hypothalamic-pituitary-adrenal system in maternal separation rats. Cell Mol Neurobiol. 2011; 31:1123–27. 10.1007/s10571-011-9718-x 21643998 PMC11498392

[33] De Kloet ER, Vreugdenhil E, Oitzl MS, Joëls M. Brain corticosteroid receptor balance in health and disease. Endocr Rev. 1998; 19:269–301. 10.1210/edrv.19.3.0331 9626555

[34] Juruena MF. Early-life stress and HPA axis trigger recurrent adulthood depression. Epilepsy Behav. 2014; 38:148–59. 10.1016/j.yebeh.2013.10.020 24269030

[35] Xue X, Shao S, Wang W, Shao F. Maternal separation induces alterations in reversal learning and brain-derived neurotrophic factor expression in adult rats. Neuropsychobiology. 2013; 68:243–49. 10.1159/000356188 24280707

[36] Ohta KI, Suzuki S, Warita K, Kaji T, Kusaka T, Miki T. Prolonged maternal separation attenuates BDNF-ERK signaling correlated with spine formation in the hippocampus during early brain development. J Neurochem. 2017; 141:179–94. 10.1111/jnc.13977 28178750

[37] Jiang Z, Zhu Z, Zhao M, Wang W, Li H, Liu D, Pan F. H3K9me2 regulation of BDNF expression in the hippocampus and medial prefrontal cortex is involved in the depressive-like phenotype induced by maternal separation in male rats. Psychopharmacology (Berl). 2021; 238:2801–13. 10.1007/s00213-021-05896-7 34328517

[38] Tenkumo C, Ohta KI, Suzuki S, Warita K, Irie K, Teradaya S, Kusaka T, Kanenishi K, Hata T, Miki T. Repeated maternal separation causes transient reduction in BDNF expression in the medial prefrontal cortex during early brain development, affecting inhibitory neuron development. Heliyon. 2020; 6:e04781. 10.1016/j.heliyon.2020.e04781 32923721 PMC7475105

[39] Zhang X, Li H, Sun H, Jiang Y, Wang A, Kong Y, Sun X, Zhu G, Li Q, Du Z, Sun H, Sun L. Effects of BDNF Signaling on Anxiety-Related Behavior and Spatial Memory of Adolescent Rats in Different Length of Maternal Separation. Front Psychiatry. 2020; 11:709. 10.3389/fpsyt.2020.00709 32793001 PMC7391957

[40] Park H, Poo MM. Neurotrophin regulation of neural circuit development and function. Nat Rev Neurosci. 2013; 14:7–23. 10.1038/nrn3379 23254191

[41] Huang H, Wang Q, Guan X, Zhang X, Zhang Y, Cao J, Li X. Effects of enriched environment on depression and anxiety-like behavior induced by early life stress: A comparison between different periods. Behav Brain Res. 2021; 411:113389. 10.1016/j.bbr.2021.113389 34058267

[42] Hoshaw BA, Malberg JE, Lucki I. Central administration of IGF-I and BDNF leads to long-lasting antidepressant-like effects. Brain Res. 2005; 1037:204–08. 10.1016/j.brainres.2005.01.007 15777771

[43] Björkholm C, Monteggia LM. BDNF - a key transducer of antidepressant effects. Neuropharmacology. 2016; 102:72–79. 10.1016/j.neuropharm.2015.10.034 26519901 PMC4763983

[44] Autry AE, Adachi M, Nosyreva E, Na ES, Los MF, Cheng PF, Kavalali ET, Monteggia LM. NMDA receptor blockade at rest triggers rapid behavioural antidepressant responses. Nature. 2011; 475:91–95. 10.1038/nature10130 21677641 PMC3172695

[45] Suzuki K, Monteggia LM. The role of eEF2 kinase in the rapid antidepressant actions of ketamine. Adv Pharmacol. 2020; 89:79–99. 10.1016/bs.apha.2020.04.005 32616215

[46] Kinnally EL, Feinberg C, Kim D, Ferguson K, Leibel R, Coplan JD, John Mann J. DNA methylation as a risk factor in the effects of early life stress. Brain Behav Immun. 2011; 25:1548–53. 10.1016/j.bbi.2011.05.001 21600281 PMC3191272

[47] Ouellet-Morin I, Wong CC, Danese A, Pariante CM, Papadopoulos AS, Mill J, Arseneault L. Increased serotonin transporter gene (SERT) DNA methylation is associated with bullying victimization and blunted cortisol response to stress in childhood: a longitudinal study of discordant monozygotic twins. Psychol Med. 2013; 43:1813–23. 10.1017/S0033291712002784 23217646 PMC4231789

[48] Baek SS, Jun TW, Kim KJ, Shin MS, Kang SY, Kim CJ. Effects of postnatal treadmill exercise on apoptotic neuronal cell death and cell proliferation of maternal-separated rat pups. Brain Dev. 2012; 34:45–56. 10.1016/j.braindev.2011.01.011 21353411

[49] Jahng JW. An animal model of eating disorders associated with stressful experience in early life. Horm Behav. 2011; 59:213–20. 10.1016/j.yhbeh.2010.11.010 21093444

[50] Wankerl M, Miller R, Kirschbaum C, Hennig J, Stalder T, Alexander N. Effects of genetic and early environmental risk factors for depression on serotonin transporter expression and methylation profiles. Transl Psychiatry. 2014; 4:e402. 10.1038/tp.2014.37 24937096 PMC4080318

[51] Hemmerle AM, Herman JP, Seroogy KB. Stress, depression and Parkinson's disease. Exp Neurol. 2012; 233:79–86. 10.1016/j.expneurol.2011.09.035 22001159 PMC3268878

[52] Amiri S, Amini-Khoei H, Mohammadi-Asl A, Alijanpour S, Haj-Mirzaian A, Rahimi-Balaei M, Razmi A, Olson CO, Rastegar M, Mehdizadeh M, Zarrindast MR. Involvement of D1 and D2 dopamine receptors in the antidepressant-like effects of selegiline in maternal separation model of mouse. Physiol Behav. 2016; 163:107–14. 10.1016/j.physbeh.2016.04.052 27143252

[53] Réus GZ, Fernandes GC, de Moura AB, Silva RH, Darabas AC, de Souza TG, Abelaira HM, Carneiro C, Wendhausen D, Michels M, Pescador B, Dal-Pizzol F, Macêdo DS, Quevedo J. Early life experience contributes to the developmental programming of depressive-like behaviour, neuroinflammation and oxidative stress. J Psychiatr Res. 2017; 95:196–207. 10.1016/j.jpsychires.2017.08.020 28886447

[54] Majidi J, Kosari-Nasab M, Salari AA. Developmental minocycline treatment reverses the effects of neonatal immune activation on anxiety- and depression-like behaviors, hippocampal inflammation, and HPA axis activity in adult mice. Brain Res Bull. 2016; 120:1–13. 10.1016/j.brainresbull.2015.10.009 26521068

[55] Maes M, Meltzer HY, Bosmans E, Bergmans R, Vandoolaeghe E, Ranjan R, Desnyder R. Increased plasma concentrations of interleukin-6, soluble interleukin-6, soluble interleukin-2 and transferrin receptor in major depression. J Affect Disord. 1995; 34:301–09. 10.1016/0165-0327(95)00028-l 8550956

[56] Dowlati Y, Herrmann N, Swardfager W, Liu H, Sham L, Reim EK, Lanctôt KL. A meta-analysis of cytokines in major depression. Biol Psychiatry. 2010; 67:446–57. 10.1016/j.biopsych.2009.09.033 20015486

[57] Udina M, Castellví P, Moreno-España J, Navinés R, Valdés M, Forns X, Langohr K, Solà R, Vieta E, Martín-Santos R. Interferon-induced depression in chronic hepatitis C: a systematic review and meta-analysis. J Clin Psychiatry. 2012; 73:1128–38. 10.4088/JCP.12r07694 22967776

[58] Kappelmann N, Lewis G, Dantzer R, Jones PB, Khandaker GM. Antidepressant activity of anti-cytokine treatment: a systematic review and meta-analysis of clinical trials of chronic inflammatory conditions. Mol Psychiatry. 2018; 23:335–43. 10.1038/mp.2016.167 27752078 PMC5794896

[59] Hashioka S, Klegeris A, Monji A, Kato T, Sawada M, McGeer PL, Kanba S. Antidepressants inhibit interferon-gamma-induced microglial production of IL-6 and nitric oxide. Exp Neurol. 2007; 206:33–42. 10.1016/j.expneurol.2007.03.022 17481608

[60] Dutcher EG, Pama EAC, Lynall ME, Khan S, Clatworthy MR, Robbins TW, Bullmore ET, Dalley JW. Early-life stress and inflammation: A systematic review of a key experimental approach in rodents. Brain Neurosci Adv. 2020; 4:2398212820978049. 10.1177/2398212820978049 33447663 PMC7780197

[61] Danese A, Moffitt TE, Pariante CM, Ambler A, Poulton R, Caspi A. Elevated inflammation levels in depressed adults with a history of childhood maltreatment. Arch Gen Psychiatry. 2008; 65:409–15. 10.1001/archpsyc.65.4.409 18391129 PMC2923056

[62] Miller GE, Cole SW. Clustering of depression and inflammation in adolescents previously exposed to childhood adversity. Biol Psychiatry. 2012; 72:34–40. 10.1016/j.biopsych.2012.02.034 22494534 PMC3493164

[63] Danese A, Caspi A, Williams B, Ambler A, Sugden K, Mika J, Werts H, Freeman J, Pariante CM, Moffitt TE, Arseneault L. Biological embedding of stress through inflammation processes in childhood. Mol Psychiatry. 2011; 16:244–46. 10.1038/mp.2010.5 20157309 PMC4212809

[64] Carroll JE, Cohen S, Marsland AL. Early childhood socioeconomic status is associated with circulating interleukin-6 among mid-life adults. Brain Behav Immun. 2011; 25:1468–74. 10.1016/j.bbi.2011.05.016 21672624 PMC3175292

[65] Hohmann CF, Odebode G, Naidu L, Koban M. Early Life Stress Alters Adult Inflammatory Responses in a Mouse Model for Depression. Ann Psychiatry Ment Health. 2017; 5:1095. 29657960 PMC5898393

[66] Zhou L, Wu Z, Wang G, Xiao L, Wang H, Sun L, Xie Y. Long-term maternal separation potentiates depressive-like behaviours and neuroinflammation in adult male C57/BL6J mice. Pharmacol Biochem Behav. 2020; 196:172953. 10.1016/j.pbb.2020.172953 32450088

[67] Wang R, Wang W, Xu J, Liu D, Wu H, Qin X, Jiang H, Pan F. Jmjd3 is involved in the susceptibility to depression induced by maternal separation via enhancing the neuroinflammation in the prefrontal cortex and hippocampus of male rats. Exp Neurol. 2020; 328:113254. 10.1016/j.expneurol.2020.113254 32084453

[68] Sadeghi M, Peeri M, Hosseini MJ. Adolescent voluntary exercise attenuated hippocampal innate immunity responses and depressive-like behaviors following maternal separation stress in male rats. Physiol Behav. 2016; 163:177–83. 10.1016/j.physbeh.2016.05.017 27184238

[69] Wang Q, Dong X, Wang Y, Liu M, Sun A, Li N, Lin Y, Geng Z, Jin Y, Li X. Adolescent escitalopram prevents the effects of maternal separation on depression- and anxiety-like behaviours and regulates the levels of inflammatory cytokines in adult male mice. Int J Dev Neurosci. 2017; 62:37–45. 10.1016/j.ijdevneu.2017.07.007 28778811

[70] Han Y, Zhang L, Wang Q, Zhang D, Zhao Q, Zhang J, Xie L, Liu G, You Z. Minocycline inhibits microglial activation and alleviates depressive-like behaviors in male adolescent mice subjected to maternal separation. Psychoneuroendocrinology. 2019; 107:37–45. 10.1016/j.psyneuen.2019.04.021 31078757

[71] Dallé E, Daniels WM, Mabandla MV. Fluvoxamine maleate normalizes striatal neuronal inflammatory cytokine activity in a Parkinsonian rat model associated with depression. Behav Brain Res. 2017; 316:189–96. 10.1016/j.bbr.2016.08.005 27569183

[72] Mohajeri MH, La Fata G, Steinert RE, Weber P. Relationship between the gut microbiome and brain function. Nutr Rev. 2018; 76:481–96. 10.1093/nutrit/nuy009 29701810

[73] Cruz-Pereira JS, Rea K, Nolan YM, O'Leary OF, Dinan TG, Cryan JF. Depression's Unholy Trinity: Dysregulated Stress, Immunity, and the Microbiome. Annu Rev Psychol. 2020; 71:49–78. 10.1146/annurev-psych-122216-011613 31567042

[74] Maes M, Kubera M, Leunis JC, Berk M. Increased IgA and IgM responses against gut commensals in chronic depression: further evidence for increased bacterial translocation or leaky gut. J Affect Disord. 2012; 141:55–62. 10.1016/j.jad.2012.02.023 22410503

[75] Wang H, Lee IS, Braun C, Enck P. Effect of Probiotics on Central Nervous System Functions in Animals and Humans: A Systematic Review. J Neurogastroenterol Motil. 2016; 22:589–605. 10.5056/jnm16018 27413138 PMC5056568

[76] Labouesse MA, Langhans W, Meyer U. Long-term pathological consequences of prenatal infection: beyond brain disorders. Am J Physiol Regul Integr Comp Physiol. 2015; 309:R1–12. 10.1152/ajpregu.00087.2015 25924881

[77] Dong TS, Gupta A. Influence of Early Life, Diet, and the Environment on the Microbiome. Clin Gastroenterol Hepatol. 2019; 17:231–42. 10.1016/j.cgh.2018.08.067 30196160 PMC6422042

[78] De Palma G, Blennerhassett P, Lu J, Deng Y, Park AJ, Green W, Denou E, Silva MA, Santacruz A, Sanz Y, Surette MG, Verdu EF, Collins SM, Bercik P. Microbiota and host determinants of behavioural phenotype in maternally separated mice. Nat Commun. 2015; 6:7735. 10.1038/ncomms8735 26218677

[79] Moussaoui N, Jacobs JP, Larauche M, Biraud M, Million M, Mayer E, Taché Y. Chronic Early-life Stress in Rat Pups Alters Basal Corticosterone, Intestinal Permeability, and Fecal Microbiota at Weaning: Influence of Sex. J Neurogastroenterol Motil. 2017; 23:135–43. 10.5056/jnm16105 27829577 PMC5216644

[80] Kelly CR, Khoruts A, Staley C, Sadowsky MJ, Abd M, Alani M, Bakow B, Curran P, McKenney J, Tisch A, Reinert SE, Machan JT, Brandt LJ. Effect of Fecal Microbiota Transplantation on Recurrence in Multiply Recurrent Clostridium difficile Infection: A Randomized Trial. Ann Intern Med. 2016; 165:609–16. 10.7326/M16-0271 27547925 PMC5909820

[81] Eckburg PB, Bik EM, Bernstein CN, Purdom E, Dethlefsen L, Sargent M, Gill SR, Nelson KE, Relman DA. Diversity of the human intestinal microbial flora. Science. 2005; 308:1635–38. 10.1126/science.1110591 15831718 PMC1395357

[82] Naseribafrouei A, Hestad K, Avershina E, Sekelja M, Linløkken A, Wilson R, Rudi K. Correlation between the human fecal microbiota and depression. Neurogastroenterol Motil. 2014; 26:1155–62. 10.1111/nmo.12378 24888394

[83] Jiang H, Ling Z, Zhang Y, Mao H, Ma Z, Yin Y, Wang W, Tang W, Tan Z, Shi J, Li L, Ruan B. Altered fecal microbiota composition in patients with major depressive disorder. Brain Behav Immun. 2015; 48:186–94. 10.1016/j.bbi.2015.03.016 25882912

[84] Valles-Colomer M, Falony G, Darzi Y, Tigchelaar EF, Wang J, Tito RY, Schiweck C, Kurilshikov A, Joossens M, Wijmenga C, Claes S, Van Oudenhove L, Zhernakova A, et al. The neuroactive potential of the human gut microbiota in quality of life and depression. Nat Microbiol. 2019; 4:623–32. 10.1038/s41564-018-0337-x 30718848

[85] Pusceddu MM, El Aidy S, Crispie F, O'Sullivan O, Cotter P, Stanton C, Kelly P, Cryan JF, Dinan TG. Correction: N-3 Polyunsaturated Fatty Acids (PUFAs) Reverse the Impact of Early-Life Stress on the Gut Microbiota. PLoS One. 2015; 10:e0142228. 10.1371/journal.pone.0142228 26426902 PMC4591340

[86] Moya-Pérez A, Perez-Villalba A, Benítez-Páez A, Campillo I, Sanz Y. Bifidobacterium CECT 7765 modulates early stress-induced immune, neuroendocrine and behavioral alterations in mice. Brain Behav Immun. 2017; 65:43–56. 10.1016/j.bbi.2017.05.011 28512033

[87] Fukui H, Oshima T, Tanaka Y, Oikawa Y, Makizaki Y, Ohno H, Tomita T, Watari J, Miwa H. Effect of probiotic Bifidobacterium bifidum G9-1 on the relationship between gut microbiota profile and stress sensitivity in maternally separated rats. Sci Rep. 2018; 8:12384. 10.1038/s41598-018-30943-3 30120330 PMC6098190

[88] Desbonnet L, Garrett L, Clarke G, Kiely B, Cryan JF, Dinan TG. Effects of the probiotic Bifidobacterium infantis in the maternal separation model of depression. Neuroscience. 2010; 170:1179–88. 10.1016/j.neuroscience.2010.08.005 20696216

[89] Liu YW, Liu WH, Wu CC, Juan YC, Wu YC, Tsai HP, Wang S, Tsai YC. Psychotropic effects of Lactobacillus plantarum PS128 in early life-stressed and naïve adult mice. Brain Res. 2016; 1631:1–12. 10.1016/j.brainres.2015.11.018 26620542

[90] Liao JF, Hsu CC, Chou GT, Hsu JS, Liong MT, Tsai YC. *Lactobacillus paracasei* PS23 reduced early-life stress abnormalities in maternal separation mouse model. Benef Microbes. 2019; 10:425–36. 10.3920/BM2018.0077 30882243

[91] Wei CL, Wang S, Yen JT, Cheng YF, Liao CL, Hsu CC, Wu CC, Tsai YC. Antidepressant-like activities of live and heat-killed Lactobacillus paracasei PS23 in chronic corticosterone-treated mice and possible mechanisms. Brain Res. 2019; 1711:202–13. 10.1016/j.brainres.2019.01.025 30684456

